# The relationship between CD4^+^CD25^+^CD127^- ^regulatory T cells and inflammatory response and outcome during shock states

**DOI:** 10.1186/cc8876

**Published:** 2010-02-15

**Authors:** François Hein, Frédéric Massin, Aurélie Cravoisy-Popovic, Damien Barraud, Bruno Levy, Pierre-Edouard Bollaert, Sébastien Gibot

**Affiliations:** 1 Service de réanimation médicale, Hôpital Central, CHU de Nancy, 29 Bld du Mal de Lattre de Tassigny, Nancy, 54000, France; 2 Laboratoire d'immunologie, Hôpital Brabois, CHU de Nancy, Avenue de la Foret de Haye, Vandoeuvre-les-Nancy, 54500, France; 3 Groupe Choc, contrat AVENIR INSERM, Faculté de Médecine, Nancy Université, Avenue de la Foret de Haye, Vandoeuvre-les-Nancy, 54500, France

## Abstract

**Introduction:**

Although regulatory T lymphocytes (Tregs) have a pivotal role in preventing autoimmune diseases and limiting chronic inflammatory conditions, they may also block beneficial immune responses by preventing sterilizing immunity to certain pathogens.

**Methods:**

To determine whether naturally occurring Treg cells have a role in inflammatory response and outcome during shock state we conducted an observational study in two adult ICUs from a university hospital. Within 12 hours of admission, peripheral whole blood was collected for the measurement of cytokines and determination of lymphocyte count. Sampling was repeated at day three, five and seven. Furthermore, an experimental septic shock was induced in adult Balb/c mice through caecal ligation and puncture.

**Results:**

Forty-three patients suffering from shock (26 septic, 17 non septic), and 7 healthy volunteers were included. The percentage of Tregs increased as early as 3 days after the onset of shock, while their absolute number remained lower than in healthy volunteers. A similar pattern of Tregs kinetics was found in infected and non infected patients. Though there was an inverse correlation between severity scores and Tregs percentage, the time course of Tregs was similar between survivors and non survivors. No relation between Tregs and cytokine concentration was found. In septic mice, although there was a rapid increase in Treg cells subset among splenocytes, antibody-induced depletion of Tregs before the onset of sepsis did not alter survival.

**Conclusions:**

These data argue against a determinant role of Tregs in inflammatory response and outcome during shock states.

## Introduction

Sepsis syndrome remains the most common cause of mortality in ICUs, causing about 750,000 deaths annually in the USA [1,2].

Despite this, our knowledge of the underlying process of sepsis is still unclear, and this is particularly obvious from an immunological standpoint. Indeed, after having considered for years the host response to severe infection only as a stormy inflammatory reaction, it appears now that anti-inflammatory components are released at the same time as pro-inflammatory mediators and the net result of these two arms of immune response determines the patient's phenotype: overwhelming inflammation with early death, delicate balance between pro- and anti-inflammation with progressive recovering, or exaggerated anti-inflammation predisposing to superinfections [3,4].

The later phenotype has been called 'immune paralysis' or 'leukocyte reprogramming' and is characterized by a lymphocytes' anergy, apoptosis, and a reduced capacity to present antigens [5]. Several mechanisms may partly explain this phenomenon such as an increased production of anti-inflammatory cytokines IL-10 and transforming growth factor (TGF)-β [3].

More recently, several groups have implicated an active lymphoid suppressor cell population: the naturally occurring regulatory T cells (Tregs) [5,6]. Although these CD4^+^CD25^+ ^cells make up only a small fraction of the T-lymphocyte population, they are potent inhibitors of immune response [7], and are now widely regarded as the primary mediators of peripheral tolerance, able to maintain immune homeostasis, to prevent autoimmunity, and to modulate inflammation induced by pathogens and environmental insults [5].

From a functional perspective, the suppressive mechanisms used by Tregs can be divided into four groups: suppression by inhibitory cytokines (such as IL-10 and TGF-β) and cell-to-cell contact, suppression by cytolysis through the secretion of granzymes, suppression by metabolic disruption, and suppression by the modulation of dendritic cells maturation or function (through cell-surface molecules such as cytotoxic T lymphocyte antigen-4, IL-10 and TGF-β) [8].

Although Tregs have a pivotal role in preventing autoimmune diseases and limiting chronic inflammatory disorders, they may also block beneficial immune responses by preventing sterilizing immunity to certain pathogens [3,9]. As an elevated percentage of Tregs was observed during septic shock, a role of this cell subset has been suspected during this syndrome [10]. Indeed, data are scarce and conflicting: whereas Heuer and colleagues demonstrated that adoptive transfer treatment with *ex vivo*-stimulated Tregs improved sepsis mortality [11], Scumpia and colleagues found that endogenous Tregs did not play a role in sepsis mortality in mice [12].

In humans, Venet and colleagues showed that, although the percentage of Tregs increased during sepsis, absolute cell count was decreased [10]. Moreover, these authors recently found an inverse correlation between Tregs and lymphocyte proliferation capacity [13].

In the present study we report that the relative increase in Tregs appears lately during septic shock and show that this phenomenon is not specific to sepsis but also occurs during non-septic shock. We also observe that the percentage of Tregs is inversely correlated with severity at admission, although with no relation to cytokine plasma concentrations. Finally, we demonstrate that Tregs do not influence survival or cytokine production in a polymicrobial model of septic shock in mice.

## Materials and methods

### Study population

All consecutive patients newly admitted to the medical ICUs of two French teaching hospital between January 2007 and June 2007 were prospectively enrolled in the study if they were suffering from shock whatever its etiology. Septic shock was defined by the standard criteria of the American College of Chest Physicians/Society of Critical Care Medicine consensus conference [14], and non-septic shock states were defined according to definitions used by Antonelli and colleagues [15]. Patients who were immunocompromised were not enrolled (treatment with corticosteroids > 0.5 mg/kg equivalent prednisolone, bone marrow or organ transplant recipients, leukopenia (white blood cell count < 1000/mm^3^) or neutropenia (polymorphonuclear granulocyte count < 500/mm^3^), hematological malignancy or AIDS). Patients who presented with early death or discharge (within 12 hours after admission) were also excluded. Seven healthy volunteers from the laboratory served as controls: four men, three women, mean age 37.5 ± 3.6 years, without chronic or acute diseases. Approvals from the Comité de Protection des Personnes-EST III and the ethical committee of the Société de Réanimation de Langue Française were obtained, and the informed consents of the patients and volunteers were sought prior to inclusion.

Upon admission to the ICU, the following data were recorded for each patient: age; sex; severity of underlying medical condition according to the criteria of McCabe and Jackson [16]; Simplified Acute Physiology Score (SAPS) II [17]; Sepsis-related Organ Failure Assessment (SOFA) score [18]; reason for admission into the ICU; principal diagnosis; vital signs; respiratory parameters; routine blood tests and microbiological culture results. Survival or death in the ICU was assessed during a follow-up period of up to 28 days.

### Blood sampling

Within 12 hours upon admission, peripheral whole blood was collected on citrated tubes. Sampling was repeated at day three, five and seven provided that the patient was still in the ICU.

### Flow cytometry

Flow cytometry analyses were conducted on a Coulter Cytomics FC500 cytometer (Beckman-Coulter, Hialeah, FL, USA). All the monoclonal antibodies and the isotypic controls (except anti-Foxp3) used were from Immunotech (Marseille, France): PE-Texas Red (ECD)-labelled anti-CD3, fluorescein isothiocyanate (FITC)-labelled anti-CD14, phycoerythrin (PE)-labelled anti-HLA-DR (clone IM 1639), FITC-labelled anti-CD3, PC5-labeled anti-CD56, PC5-labelled anti-natural killer (NK) G2D, PC5-labeled anti-CD4, FITC-labelled anti-CD25 and PE-labelled anti-CD127; the Foxp3 monoclonal antibody was from eBiosciences (San Diego, CA, USA). After red blood cells lysis (Q-prep system, Beckman Coulter, Hialeah, FL, USA), naturally occurring Tregs were characterized by the expression of CD4 and CD25 and the lack of expression of CD127. We also characterized the number of monocytes and NK cells based upon the expression of the usual markers (CD45, CD14, HLA-DR, CD16, and NKG2D, respectively). Results are expressed as percentages of the CD4^+ ^lymphocyte population and as number of cells per microliter of whole blood.

### Cytokines plasma concentrations

Plasma concentrations of IL-2, IL-4, IL-5, IL-10, interferon (INF) γ and TNFα were quantified through fluorescence activated cell sorting (FACS) by the use of human Th1/Th2 CBA kit (BD Biosciences, San Diego, CA, USA) according to the recommendations of the manufacturer. Intra- and inter-assay coefficients of variation were less than 8%. Concentration of TGFβ was determined by ELISA (R&D Systems, Lille, France).

### Polymicrobial sepsis in mice

All experiments were approved by the Institutional Animal Care and Use Committee. Adult (5 to 6 weeks, 20 to 23 g) male Balb/c mice were purchased from the Centre d'élevage Dépré (Saint-Doulchard, France).

For induction of polymicrobial sepsis, mice were anesthetized by intraperitoneal administration of ketamine and xylazine in 0.2 ml sterile pyrogen-free saline. The cecum was exposed through a 1.0 cm abdominal midline incision and subjected to ligation of the distal half followed by two punctures with a 21G needle. A small amount of stool was expelled from the punctures to ensure patency. The cecum was replaced into the peritoneal cavity and the abdominal incision closed in two layers. After surgery, all mice were injected subcutaneously with 0.5 ml of physiologic saline solution for fluid resuscitation and subcutaneously every 12 hours with 1.25 mg (i.e., 50 μg/g) of imipenem. A sham operation was performed by isolating the cecum without ligation or puncture.

When specified, animals were given either an intraperitoneal injection of 500 μg of affinity purified CD25 (PC61 hybridoma)-depleting antibody (BioExpress, West Lebanon, NH, USA) or an identical volume (250 μl) of sterile normal saline 72 hours before the caecal ligation and puncture (CLP). Administration of the antibody (250 μg) or normal saline was repeated two hours before the procedure.

Animals were observed for up to seven days to determine survival. For certain experiments, 4 to 6 mice were euthanized at 24, 48 or 96 hours after surgery to harvest blood or splenocytes. Among splenocytes, the Tregs were considered to be the CD4^+^CD25^+^Foxp3^+ ^cells on FACS.

At indicated times, plasma concentrations of TNF-α, IL-1β and IL-6 were measured by ELISA.

### Statistical analysis

Changes in time or between groups were analyzed by one-way analysis of variance. Comparisons between patients with septic shock, patients with non-septic shock and/or healthy volunteers were performed by using Student's *t *test as normally distributed values (Kolmogoroff-Smirnov test), Mann-Whitney *U *test, or Kruskal Wallis test when indicated. Data are expressed as mean ± standard deviation or median ± interquartile range when appropriate. Correlations were established using Spearman's test. Survival analysis (mice) was performed using the log rank test after drawing the Kaplan Meier survival curves. Analysis was completed with the Statview software (Abacus Concepts, Berkeley, CA, USA), with a two-tailed *P *value less than 0.05 considered as statistically significant.

## Results

From January to June 2007, 43 consecutive patients suffering from shock were included. There were 26 septic shocks (10 of pulmonary origin, 4 peritonitis, 2 endocarditis, 3 urinary tract infections and 7 bacteremia of unknown origin) and 17 non-septic shocks (10 cardiogenic, 3 hemorrhagic, and 4 toxic). Characteristics of the patient population are shown in Table 1. The high mortality (42%) was expected regarding the SOFA and SAPS II severity scores.

**Table 1:** Characteristics of the studied population

Characteristic*	Septic shock patients (n = 26)	Non-septic shock patients (n = 17)	*P *value
Age, years	67 (6)	59 (17)	NS
Sex^†^			NS
Male	19 (73)	11 (65)	
Female	7 (27)	6 (35)	
McCabe and Jackson criteria	1 (1)	1 (1)	NS
SAPS II score	70 (22)	73 (24)	NS
SOFA score	13 (5)	11 (5)	NS
Mean arterial pressure, mmHg	72 (12)	71 (12)	NS
Catecholamines^†^	26 (100)	17 (100)	NS
Mechanical ventilation^†^	26 (100)	16 (93)	NS
Corticosteroids^†^	16 (62)	3 (18)	0.01
Extra-renal support^†^	10 (38)	7 (40)	NS
Arterial lactate (mmol/L)	5 (4)	6 (4)	NS
C-reactive protein, mg/L	178 (110)	54 (49)	0.001
Procalcitonin (ng/mL)	34 (42)	1.4 (1.8)	< 0.001
Neutrophils (cells/μL)	9806 (6891)	15077 (4178)	0.009
Lymphocytes (cells/μL)	684 (647)	2305 (2719)	0.001
Monocytes (cells/μL)	894 (862)	1000 (772)	NS
Mortality rate^†^	11 (42)	7 (41)	NS

*Values are expressed as mean (standard deviation). *P *values are for the comparison of septic shock vs non-septic shock patients.

NS, not significnat; SAPS, Simplified Acute Physiology Score; SOFA, Sepsis-related Organ Failure Assessment.

^†^Values are expressed as number (percentage).

The septic shock patients had lower neutrophils and lymphocytes counts, higher plasma concentrations of C-reactive protein and procalcitonin as compared with non-septic patients.

### Kinetics of Tregs

**Figure 1:**
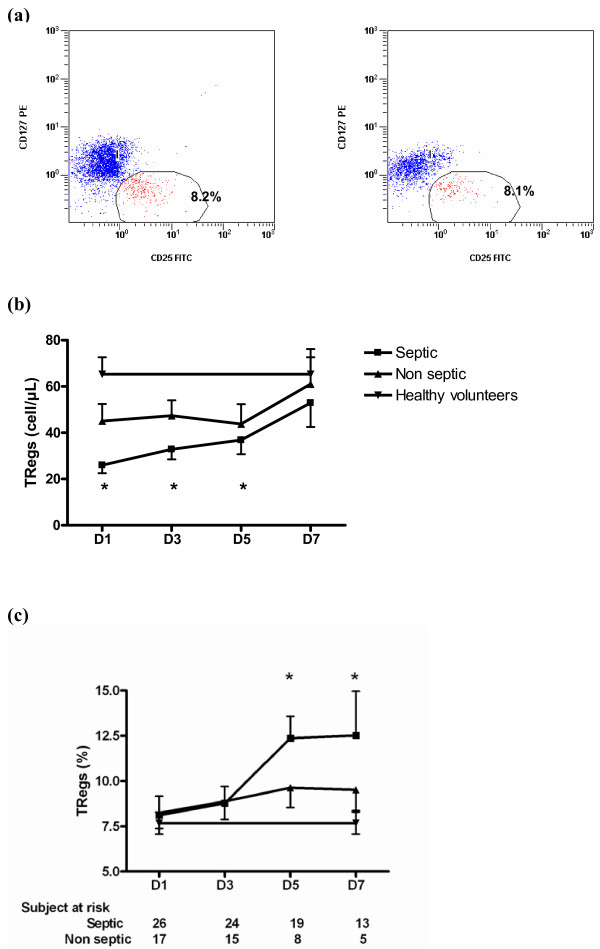
**Treg cells kinetics during shock**. Regulatory T lymphocytes (Treg) cells are defined as CD4^+^CD25^+^CD127^- ^lymphocytes. Percentages of Tregs among CD4^+ ^lymphocytes, as well as absolute count are determined in shock patients within 12 hours of admission, and then at days three, five and seven. Seven healthy volunteers served as controls. **(a) **Examples of Tregs percentage determination in a healthy volunteer (left panel) and a septic shock patient on admission (right panel). **(b) **Time-course of Tregs absolute count and **(c) **Percentage in patients with shock. * *P *< 0.03 patients vs. healthy volunteers.

Naturally occurring Tregs were defined as CD4^+^CD25^+^CD127^- ^cells (Figure 1a).

At day one, the absolute number of Tregs was lower in patients than in healthy volunteers (Figure 1b and Table 2) without any difference between the two groups of patients. When expressed as a percentage of CD4^+ ^lymphocytes, no differences were observed between patients and volunteers on admission (Figure 1c and Table 2).

**Table 2:** The sub-populations of lymphocytes on admission

	Septic shock (n = 26)	Non septic shock (n = 17)	Healthy volunteers (n = 7)	*P *value
CD4^+ ^lymphocytes (cells/μL)*	343 (230)	760 (926)	890 (384)	NS
Tregs (cells/μL)^†^	27 (13-33)	43 (22-66)	65 (45-72)	0.03
Tregs (% of CD4+)	8,2 (1)	8.1 (1)	8 (2)	NS
NK cells (cells/μL)^†^	55 (32-85)	94 (27-228)	213 (115-294)	0.03
NK cells (% of lymphocytes)	5.4 (0.6)	10.2 (0.8)	11.6 (0.8)	NS

*Values are expressed as mean (standard deviation).

^†^Values are expressed as median (interquartile range).

*P *values are for the comparison between the three groups.

NK, natural killer; NS, not significant; Tregs, regulatory T lymphocytes.

By day three, both Treg numbers and percentages progressively increased in both groups of patients, especially the septic ones, although there was no statistical difference between the two groups of patients (Figures 1b and 1c). At day seven, although the percentage of Tregs was higher in the septic patients than in the non-septic patients and healthy volunteers, there were no differences in terms of absolute count, reflecting the persistence of the CD4^+ ^lymphopenia in the septic shock group.

**Figure 2:**
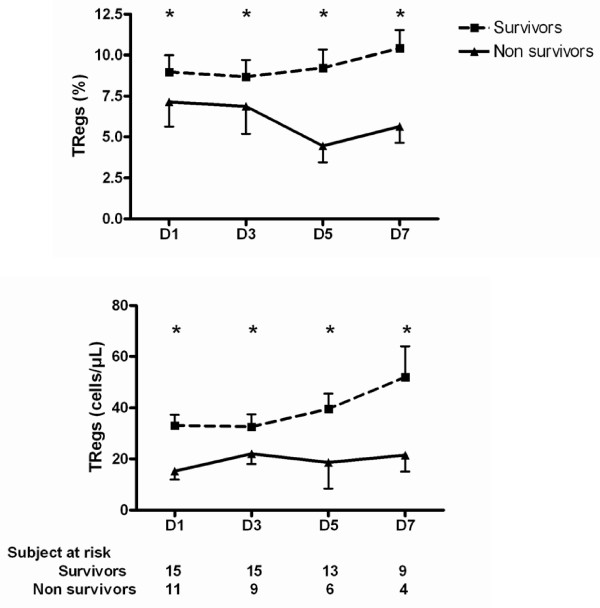
**Treg cells kinetics according to outcome in septic shock patients**. * *P *= 0.02 septic survivors vs. septic non survivors. Treg, regulatory T lymphocytes.

We next sought to evaluate the relation between Treg kinetics and survival. Considering the whole group of patients, or those suffering from a non-septic shock, there were no differences between survivors and non-survivors in terms of percentage or number of Tregs. By contrast, within the septic shock patients, those surviving had constantly higher Tregs counts and percentages than non-survivors (Figure 2).

### Correlation between Tregs, severity and cytokines plasma concentrations

**Figure 3:**
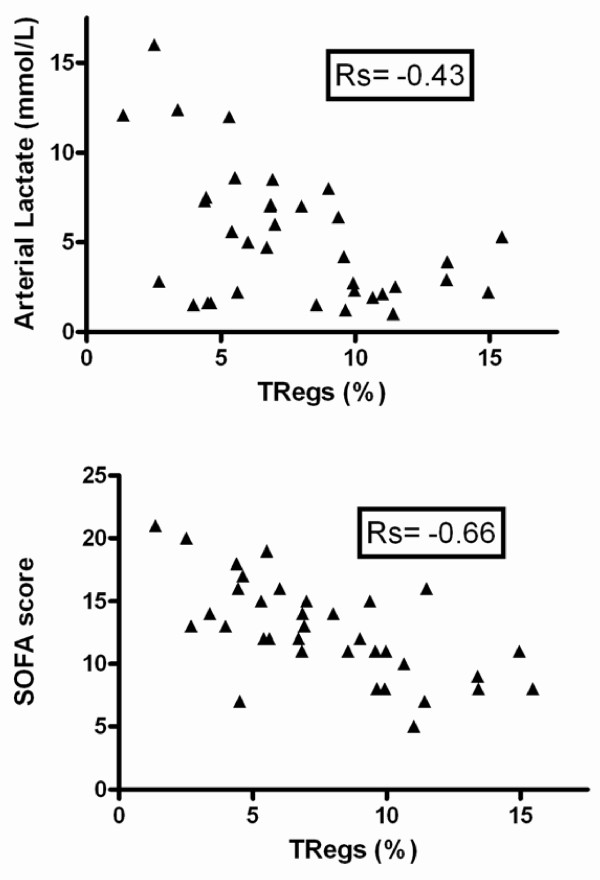
**Correlation between Tregs and severity on admission**. Regulatory T lymphocytes (Tregs) percentages are inversely correlated to arterial lactate concentration (*P *= 0.01) and Sepsis-related Organ Failure Assessment (SOFA) score (*P *= 0.0001).

Considering the correlation between Tregs and outcome, at least in septic patients, we evaluated the relation between these cells and severity. Indeed, there was an inverse correlation between the percentage of Tregs and SAPS II (not shown), SOFA score or arterial lactate level (Figure 3).

By contrast, we were unable to find any correlation between Tregs and plasma cytokine (IL-2, IL-4, IL-5, IL-10, INFγ, TNFα and TGF-β) concentrations, either taken individually or expressed as Th1/Th2 ratios (data not shown). This absence of correlation was found in septic as well as non-septic patients.

### NK cells kinetics

NK cells have been advocated as an important component influencing outcome during septic shock [18].

**Figure 4:**
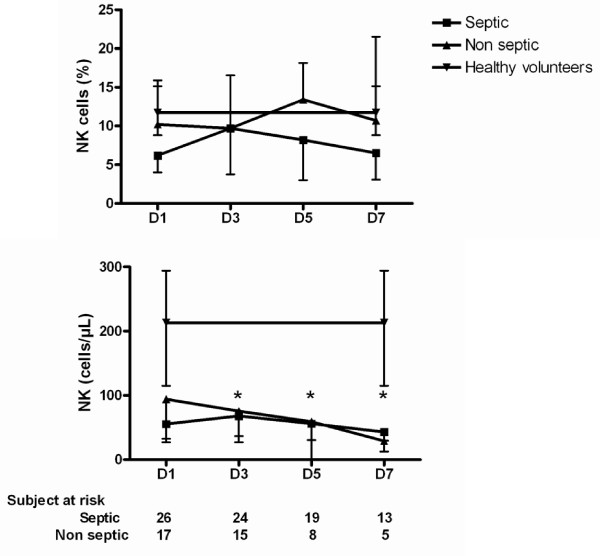
**NK cells kinetics during shock**. Time-course of natural killer (NK) cells absolute count and percentage (among total lymphocytes) in patients with shock. * *P *< 0.01 patients vs. healthy volunteers.

We observed that, although the percentage of NK cells was not different between patients and healthy volunteers throughout the study period, patients with shock presented with constantly decreased NK absolute numbers (Figure 4). As we observed for the Treg counts, there were no differences between septic and non-septic shock patients, or between survivors and non-survivors. There was no correlation between the percentages or absolute counts of Tregs and NK cells.

### Polymicrobial sepsis in mice

**Figure 5:**
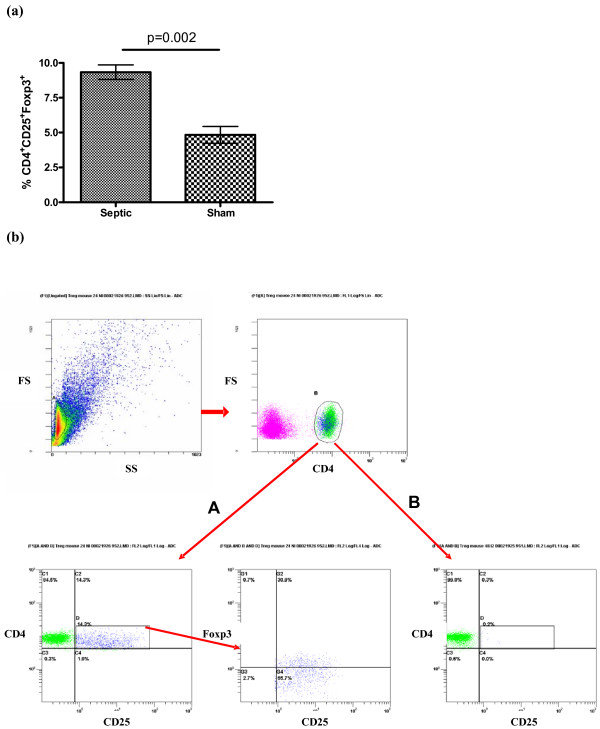
**Sepsis increases Tregs percentage in mice**. Twenty-four hours after caecal ligation and puncture (CLP), proportion of regulatory T lymphocytes (Treg) cells among splenocytes (CD4^+^CD25^+^Foxp3^+ ^cells) is determined (n = 6 per group) **(a)**. In some experiments, Tregs were depleted by using anti-CD25 antibody prior to sepsis induction. Depletion was highly effective with less than 1% of Tregs remaining 24 hours after the **(b, B) **last antibody injection as compared with **(b, A) **control animals.

To get further insight in to the role of Tregs during septic shock, we used a CLP-induced model of peritonitis in mice. We first observed that as early as 24 hours after surgery, there was an increase in the percentage of Tregs among splenocytes (CD4^+^CD25^+^Foxp3^+ ^cells) of septic mice as compared with the sham-operated ones (Figure 5).

**Figure 6:**
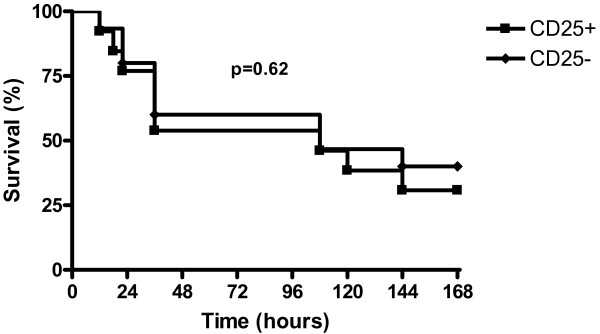
**Survival according to the regulatory T lymphocytes status in septic mice**. Antibody-mediated depletion of CD25^+ ^cells does not alter survival during caecal ligation and puncture (n = 20 per group, *P *= 0.62).

We next sought to investigate the influence of Tregs on survival. We depleted animals of CD25^+ ^cells by using repeated injection of anti-CD25 antibody. Depletion was highly effective with less than 0.2% of CD25^+ ^cells left 24 hours after the last antibody injection (Figure 6). This Treg depletion persisted at day four but was not checked thereafter.

**Figure 7:**
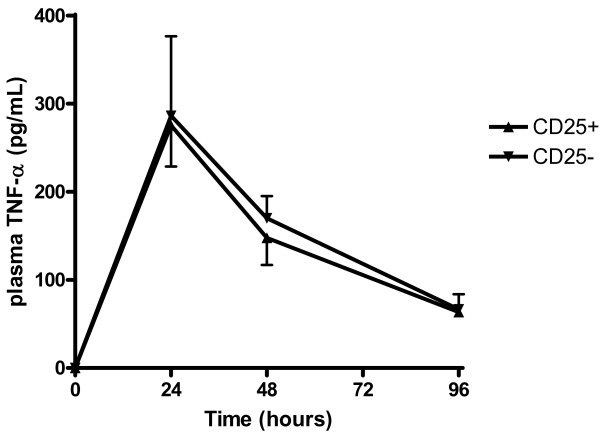
**TNF-α plasma concentration according to the regulatory T lymphocytes status in septic mice**. Antibody-mediated depletion of CD25^+ ^cells does not influence TNF-α plasma concentration during caecal ligation and puncture (n = 6 per group).

Animal survival was not affected by the CD25^+ ^cells status with 40% of CD25-depleted mice surviving as compared with 31% for the non-depleted group (*P *= 0.62; Figure 7).

Finally, plasma concentrations of TNF-α (Figure 8), IL-1β or IL-6 (not shown) were not different between the two groups of mice.

## Discussion

There is now enough evidence that morbidity and mortality occurring during septic, as well as non-septic, shock may be due to the effect of distinct mechanisms over time: in addition to an overwhelming pro-inflammatory immune response, concomitant counter-inflammatory mechanisms develop that may be responsible for 'immune paralysis' [3]. Although naturally occurring Tregs have been found to play major roles in a wide range of disorders, such as tumour progression, inflammation or transplantation tolerance [4,7,9], their implication during shock states is unclear.

In agreement with the findings of Monneret and colleagues [10,19], we observed that the percentage of Tregs increased as early as three days after the onset of shock, while their absolute number remained lower than in healthy volunteers. This phenomenon has been explained by Venet and colleagues by a decrease of the CD4^+^CD25^- ^lymphocytes subset [10], most probably through apoptosis. Adding to the similarities that exist between septic and non-septic shock in terms of immune derangement, we found similar patterns of Treg kinetics in infected and non-infected patients. Indeed, immune paralysis is also observed during trauma/haemorrhage or cardiogenic shock as demonstrated by a reduced ability of antigen presentation or *ex vivo *pro-inflammatory cytokine production [20]. Nevertheless, this apparent absence of difference between septic and non-septic patients may also, at least in part, be explained by the small sample size and the variability in each of the measurements.

Although we observed an inverse correlation between severity, assessed by SOFA score or arterial lactate concentration, and percentage of Tregs, the time course of the percentage or absolute number of Tregs was similar between survivors and non-survivors. Most probably, this finding may also be explained by the negative correlation between severity and CD4^+^CD25^- ^lymphocyte number (data not shown). Nevertheless, only considering the group of septic shock patients, we observed that survivors had higher percentage and absolute numbers of Tregs by day five than non-survivors. Although this finding may suggest a specific effect of Tregs in sepsis outcome, the low number of non-survivors studied at days five and seven preclude any definite conclusion.

The influence of cytokines on Treg proliferation or activation is unknown during shock states, as is the effect of Tregs on cytokine production. Here we were unable to show any correlation between percentage or absolute count of Tregs and plasma cytokine concentration (IL-2, IL-4, IL-5, IL-10, INFγ, TNFα and TGF-β), taken individually or expressed as a Th1/Th2 balance. Therefore, Tregs do not seem to modulate the inflammatory response during shock *in vivo*. Of note, this absence of relation was found in septic as well as in non-septic patients.

As NK cells have recently been advocated to play a role in sepsis outcome, we also investigated the kinetics of this cell's subset. In contrast to the findings of Giamarellos-Bourboulis and colleagues [21], we observed that the percentage of NK cells was not different between patients and healthy volunteers throughout the study period. This discrepancy may be explained by the unexpectedly low NK percentage (4.12%) found in the six healthy volunteers, and the old age of the patients (77 years) enrolled in the Greek study. Patients with shock presented with a constantly decreased NK absolute count, without any differences between septic and non-septic shock. Finally, NK cell kinetics was similar between survivors and non-survivors. Taken together, these data suggest a marginal role of NK cells on shock outcome, although the relatively small number of patients enrolled precludes definite conclusion.

To further evaluate the role of Tregs on sepsis outcome, we used a well characterized CLP model of polymicrobial sepsis in mice. In accordance with Scumpia and colleagues [12], we observed a rapid increase in Tregs subset among splenocytes in septic animals. However, despite their increased proportion, antibody-induced depletion of Tregs before the onset of sepsis did not alter survival. Again, these data are in line with those of Scumpia and colleagues [12]. The originality of the present study stems from the septic shock model we used: mice were fluid resuscitated and antibiotics given, further approaching the complex physiopathology of human sepsis. Therefore, our data are more able to be extrapolated to human septic shock. In accordance with what we observed in humans, Tregs did not appear to influence cytokine production (TNF-α, IL-1β, IL-6) in septic animals.

In contrast, our animal data seem in discordance with those reported by Heuer and colleagues [11]. Several important differences between these two studies need to be underlined. First, Heuer and colleagues performed an adoptive transfer of *in vitro *prestimulated Tregs and then it is not clear whether the protective role on survival was due to Tregs themselves or to the artificial induction of membrane/soluble mediators. Second, the model of CLP we used here appears to be more stringent and thus, a potential protective role of Tregs may have been masked by the severity of the model. Finally, although we used the classical and well-accepted anti-CD25 antibody approach to deplete Tregs, we must acknowledge that anti-CD25 is not specific for Tregs and leads to the depletion of other important cells (activated T cells). Moreover, anti-CD25 antibody may not have completely eliminated CD25^low ^Tregs.

## Conclusions

Taken together, these data argue against a determinant role of naturally occurring Tregs on inflammatory response and outcome during shock states.

## Key messages

• Percentage of Tregs increases early after the onset of shock whatever its etiology, while their absolute number remains lower than in healthy volunteers.

• Percentage of Tregs is inversely correlated to severity of shock, although the time course of percentage or absolute number of Tregs is similar between survivors and non-survivors.

• There is no correlation between percentage or absolute number of Tregs and plasma cytokine concentrations taken individually or expressed as a Th1/Th2 balance.

• In a mouse model of septic shock, antibody-induced depletion of Tregs before the onset of sepsis does not alter survival.

## Abbreviations

ELISA: enzyme-linked immunosorbent assay; FACS: fluorescent activated cell sorting; FITC: fluorescein isothiocyanate; IFN: interferon; IL: interleukin; NK: natural killer; PE: phycoerythrin; SAPS: Simplified Acute Physiology Score; SOFA: Sepsis-related Organ Failure Assessment; TGF: transforming growth factor; TNF: tumor necrosis factor; Tregs: regulatory T cells.

## Competing interests

The authors declare that they have no competing interests.

## Authors' contributions

FH and SG designed the study, enrolled patients, performed experiments, and wrote the manuscript; FM performed experiments; ACP, DB, BL and PEB enrolled patients and analyzed data. All authors read and approved the final manuscript.
